# Improved oxidative status in major abdominal surgery patients after N-acetyl cystein supplementation

**DOI:** 10.1186/1475-2891-14-4

**Published:** 2015-01-06

**Authors:** Aygun Kuyumcu, Asli Akyol, Zehra Buyuktuncer, M Mahir Ozmen, Halit Tanju Besler

**Affiliations:** Department of Nutrition and Dietetics, Faculty of Health Sciences, Hacettepe University, Ankara, Turkey; Değişim Nutrition Consultancy and Education Services, Ankara, Turkey; Department of General Surgery, Ankara Numune Training and Research Hospital, Ankara, Turkey; Department of General Surgery, Faculty of Medicine, Hacettepe University, Ankara, Turkey

**Keywords:** Major abdominal surgery, N-acetyl cystein, Plasma amino acids, Oxidant parameters

## Abstract

**Background:**

Increased levels of reactive oxygen species during and after surgery may affect inflammatory response, post-operative adhesion molecule formation, and hemodynamic stability. The glutathione redox cycle is an important regulator in oxidative stress and its reduced forms scavenge free radicals. N-acetyl cysteine, a precursor of reduced glutathione, is considered as a potentially therapeutic wide spectrum agent in clinical practice. We therefore examined whether N-acetyl cysteine improves some biochemical parameters in cancer patients undergoing major abdominal surgery.

**Methods:**

Thirty-three patients diagnosed with pancreas, stomach, rectum, colon malignancies, and undergoing major abdominal surgery at Ankara Numune Training and Research Hospital were randomly divided into two groups; control (CON) and N-acetyl cysteine (NAC). The NAC group had 1,200 mg N-acetyl cysteine starting two days before the operation day, in addition to isonitrogenous and isocaloric total parenteral nutrition of 1.2 g/kg protein, 25 kcal/kg, and 60:40 carbohydrate/fat ratio. Blood and urine samples were drawn two days before the operation, on operation day, and on the first, third, and fifth days post-operation.

**Results:**

Plasma malondialdehyde was significantly lower in the NAC group (P < 0.001). N-acetyl cysteine treatment did not affect plasma levels of vitamin A, C or E. The NAC group exhibited a higher ratio of reduced glutathione to oxidised glutathione (P = 0.019). Urinary nitrate level was also significantly lower in the NAC group (P = 0.016).

**Conclusion:**

The study demonstrated the clinical importance of N-acetyl cysteine supplementation on antioxidant parameters in abdominal surgery patients. In these patients N-acetyl cysteine and vitamin administration can be considered as an effective method for improvement of oxidative status.

## Introduction

Free radicals are important compounds that may affect progress in trauma, tissue damage, and chronic degenerative diseases [1]. Under normal physiological conditions these reactive compounds are removed by an organism’s antioxidant mechanisms [2]. However, lack of balance between oxidant and antioxidant molecules may trigger harmful effects of free radicals, especially under sub-optimal conditions such as cancer [3]. Therefore, reducing oxidative stress and supporting the antioxidant system are considered as substantial approaches in clinical practice [4, 5].

N-acetyl cysteine (NAC) is the N-acetylated form of amino acid L-cysteine and is used in the first step of glutathione (GSH) synthesis, by extracting cysteine from the N-acetylated derivative [6]. Since GSH is the fundamental thiol antioxidant of the human body and NAC provides a rate-limiting cysteine needed for glutathione synthesis, much work to date has investigated the role of NAC as an oxidative stress suppressor in various diseases [7–11]. It was shown that NAC exerts these antioxidant effects through promoting glutathione synthesis [12]. The direct free radical chelating property of NAC has also been examined, although results relating to the reactivity of NAC with superoxide anion and hydrogen peroxide have been controversial [13]. In addition to these mechanisms, reduction in leukocyte-endothelium interaction, oxidative burst of neutrophils, anti-inflammatory, and mucolytic actions were all associated with NAC [12–14].

Most of the studies that assess the effectiveness of NAC have focused on cardiac, liver, and abdominal surgery, due to its protective effects against oxidant stress contributed by surgical operations. These studies have demonstrated some beneficial effects of NAC on postoperative atrial fibrillation, postoperative adhesion formation, ischemia/reperfusion injury, and peritoneal fibrinolytic activity [14–17]. However, results have been inconsistent with those of other trials in which the influence of NAC was examined [18, 19]. Certainly, there is strong evidence to support the direct effect of NAC on antioxidant capacity of glutathione redox cycle *in vitro*[12]. Hence, it is of interest to investigate the effect of NAC treatment on related biomarkers. For this perspective, the objective of this study was to examine the effect of NAC treatment on oxidant, antioxidant, and plasma amino acid levels in major abdominal surgery patients.

## Material and methods

Thirty-three non-smoker oncology patients (18 male and 15 female) undergoing major abdominal surgery at Ankara Numune Training and Research Hospital were included in the study. Patients were diagnosed with pancreas cancer (CON, n = 3; NAC, n = 3), stomach cancer (CON, n = 10; NAC, n = 7), rectum cancer (CON, n = 3; NAC, n = 4), and colon cancer (CON, n = 1; NAC, n = 2). The exclusion criteria were as follows: emergency operation; pregnancy; breast feeding; impaired renal function; preoperative IV feeding; inability to maintain hemodynamic conditions that allowed optimal conventional resuscitation; mean arterial pressure persistently under 70 mm Hg despite inotropic support; hemotocrit values below 30% or receiving blood transfusions; unable to keep a PaO_2_ of 80 to 140 mm Hg and CO_2_ of 35 to 50 mm Hg or requiring a fractional inspired oxygen concentration (FiO_2_) of over 50; severe heart disease; or taking calcium channel antagonists, angiotensin converting enzyme inhibitors, corticosteroids, NAC, or other drugs with antioxidant activity. Patients were randomly divided into two groups: control (CON) and experimental (NAC) group. All patients received isonitrogenous and isocaloric total parenteral nutrition of 1.2 g/kg protein, 25 kcal/kg, and 60:40 carbohydrate/fat ratio. The NAC group was given 1,200 mg of NAC (300 mg in every 6 h) through total parenteral nutrition, starting from 2 days before the operation and lasting until the fifth post-operative day. This dose of NAC supplementation was considered to be clinically relevant in order to avoid pro-oxidant effects. Blood and 24 h urine samples were collected 2 days before operation (baseline), on operation day, and the first, third and fifth post-operation days. The study protocol was approved by the Minister of Health Research Ethics Committee (B100İEG0110011).

### Anthropometric measurements

Body weight and body composition of patients were measured by a Bodystat 1500 (Bodystat Douglas Isle of Man, UK). In addition to body composition, mid-upper arm circumference (MUAC) and triceps skinfold thickness (TSFT) were measured. Nutritional Risk Index (NRI) was derived as follows: NRI = (1.519 × serum albumin, g/L) + (41.7 × present/usual body weight). A NRI score of >100 indicates no risk for the patient; 97.5 to 100, mild risk; 83.5 to 97.5, moderate risk; and < 83.5, severe risk.

### Blood and urine samples

Pre-operative baseline blood samples were taken after 12 h of starvation. Following two days of NAC supplementation or control treatment, patients were operated. Operation day blood samples were taken 2 h after surgery. Post-operation day 1, 3 and 5 blood samples were taken. Twenty-four hour urine samples were collected on the same days in sterile containers and stored at -20°C. Blood samples were centrifuged and stored at -80°C. Plasma amino acid analyses were performed using a GC amino acid kit (EZ:faast).

### Blood indicators of oxidative stress and antioxidants

Reduced (GSH) and oxidized glutathione (GSSG) were determined from whole blood samples and malondialdehyde (MDA) was analyzed from plasma samples using high pressure liquid chromatography [20, 21]. Similarly, vitamin C, A, and E were assayed using high pressure liquid chromatography of plasma samples as previously described [22, 23].

### Nitrate and nitrite analysis

Urine nitrate and nitrite was determined using a spectrophotometer (UNICAM 1500) and read at an absorbance of 538 nm. Glycine–(NaOH) buffer and copper (CuSO_4_) saturated cadmium were used to reduce nitrate to nitrite [24].

### Statistical analysis

All data were analysed using the Statistical Package for Social Sciences (version 16; SPSS, Inc., Chicago, IL, USA). The effect of NAC supplementation on biochemical and antioxidant parameters was examined using ANOVA with factors of treatment and time (i.e., the day that the samples were taken [pre-operation, operation day, and the first, third, fifth days post-operation]). Tukey’s post hoc test was performed when the main time effect was significant. Values are expressed as mean values with their standard errors unless otherwise stated. P < 0.05 was considered statistically significant.

## Results

### Participants

All 33 subjects completed the study successfully. Table 1 shows the characteristics of the two study groups. Age and anthropometric measurements were similar between groups, yet BMI and body composition were different between male and female participants. NRI data indicated that all of the participants were at risk of malnutrition (Table 1). This was a mild risk for female participants of the NAC group, whereas female participants of the control group and all other male participants exhibited a moderate risk of malnutrition. Total and post-operative lengths of stay at hospital were similar between groups (Table 1).

**Table 1:** **Characteristics of the two study groups**

	Control group		NAC group	
	Male (n = 9)	Female (n = 8)	Male (n = 9)	Female (n = 7)
Age (y)	60.44 ± 4.35	57.38 ± 3.58	56.56 ± 4.05	65.29 ± 5.49
BMI (kg/m^2^)^a^	20.05 ± 1.03	25.80 ± 1.45	20.99 ± 1.18	25.11 ± 0.79
Body fat (%)^a^	32.07 ± 2.65	44.44 ± 3.38	27.60 ± 4.05	42.81 ± 3.2
Body water (%)^a^	55.00 ± 3.98	46.90 ± 2.45	59.30 ± 3.47	48.20 ± 2.45
Fat-free mass (kg)^a^	38.93 ± 2.30	33.24 ± 2.82	43.44 ± 3.46	35.96 ± 3.87
Triceps skin fold thickness^a^	6.93 ± 0.86	18.05 ± 1.45	8.27 ± 1.49	18.96 ± 2.07
Mid upper arm circumference (cm)	22.76 ± 0.84	22.80 ± 0.76	23.43 ± 1.22	23.79 ± 1.08
Nutritional risk index	92.70 ± 3.30	95.65 ± 3.68	92.41 ± 5.06	98.97 ± 2.55
Total length of stay (d)	23.11 ± 3.85	27.89 ± 3.85	30.88 ± 4.09	23.57 ± 4.37
Post-operation length of stay (d)	15.89 ± 6.15	19.44 ± 4.11	21.50 ± 5.19	11.29 ± 1.59

Data presented as mean ± SEM.

^a^Significantly different between male and female participants in all groups (P < 0.001).

### Plasma amino acid levels

**Figure 1:**
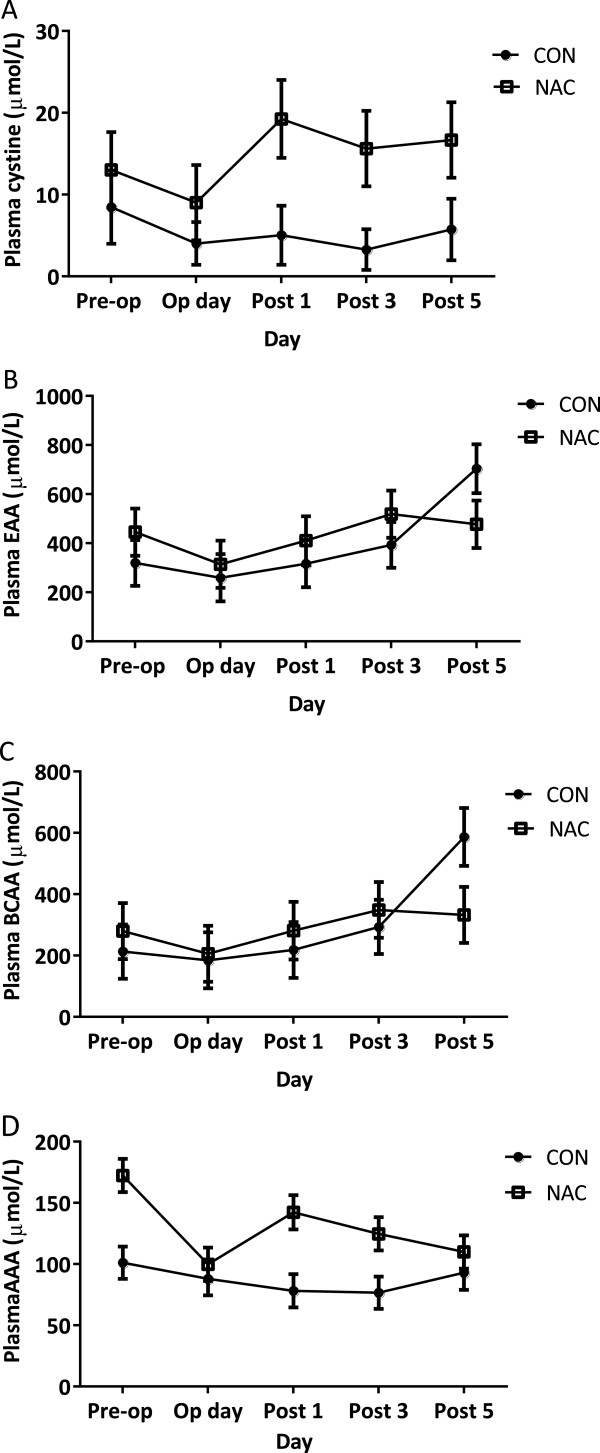
**Data presented as mean ± SEM.** CON n = 17, NAC n = 16. **A**: Plasma cystine levels. NAC group had significantly higher plasma cystine levels compared to the control group (P = 0.002). No significant effect of day was found on plasma cystine level. **B**: Plasma EAA levels. Tukey’s post-hoc test showed a significant difference between operation day and post-operational fifth day on plasma EAA levels in both study groups (P = 0.029). No significant effect of treatment was found on plasma EAA. **C**: Plasma BCAA levels. Tukey’s post-hoc test showed a significant difference between operation day and post-operational fifth day on plasma EAA levels in both study groups (P = 0.046). No significant effect of treatment was found on plasma BCAA. **D**: NAC group had significantly higher plasma AAA when compared to control group (P < 0.001). Tukey’s post-hoc test showed a significant difference between pre-operation day and operation day on plasma AAA levels in both study groups (P = 0.017).

NAC treatment (1,200 mg) resulted in significantly increased plasma cystine concentration throughout the study (5.30 ± 2.05 µmol/L, CON versus 14.71 ± 2.08 µmol/L, NAC, P = 0.002) (Figure 1A). Although there was a trend towards a rise in plasma cystine concentration during post-operational days in the NAC group, the effect of day was not significantly important in either group. Plasma essential amino acid (EAA) levels was unaffected by NAC treatment at any stage of the study (398.28 ± 42.87 µmol/L, CON versus 433.05 ± 43.38 µmol/L, NAC, P > 0.05) (Figure 1B). However, time exhibited a significant effect on plasma EAA levels since there was a significant difference in plasma EAA concentration between the operation day and post-operational fifth day in both groups (286.49 ± 68.13 µmol/L, operation day versus 590.33 ± 69.26 µmol/L, post-operational fifth day, P = 0.029). Similarly, branched chain amino acid (BCAA) concentration was not influenced by NAC treatment, although it exhibited a significant difference between the operation day and post-operational fifth day (286.49 ± 68.13 µmol/L, operation day versus 590.33 ± 69.26 µmol/L, post-operational fifth day, P = 0.046) (Figure 1C). Plasma aromatic amino acid (AAA) concentration was found to be affected by both treatment (P < 0.001) and day (P = 0.017) (Figure 1D). While plasma AAA was significantly higher in the NAC group than the control group (87.37 ± 6.04 µmol/L, CON versus 129.77 ± 6.11 µmol/L, NAC, P < 0.001); and was also significantly lower on operation day when compared to pre-operational values in both groups (136.71 ± 9.45 µmol/L, pre-operation versus 93.88 ± 9.59 µmol/L, operation day, P = 0.017).

### Oxidant and antioxidant parameters

Oxidant and antioxidant parameters of the two study groups are shown in Table 2. Plasma MDA exhibited significantly lower levels in the NAC group when compared to controls (0.44 ± 0.01 µmol/L, CON versus 0.36 ± 0.01 µmol/L, NAC, P < 0.001). This effect of NAC on plasma MDA was independent of the time of sampling, which suggests that plasma MDA concentration was not influenced by pre-, post-, or day of operation. A similar pattern was observed for urinary nitrate levels, which showed that the NAC group had significantly lower nitrate concentrations (350.73 ± 31.15 µmol/L, CON versus 242.17 ± 31.91 µmol/L, NAC, P = 0.016), irrespective of the day. The exception to this was the urinary nitrite level, which was similar between groups (10.83 ± 1.07 µmol/L, CON versus 9.30 ± 1.09 µmol/L, NAC, P > 0.05) and was not affected by sample day (Table 2).

**Figure 2:**
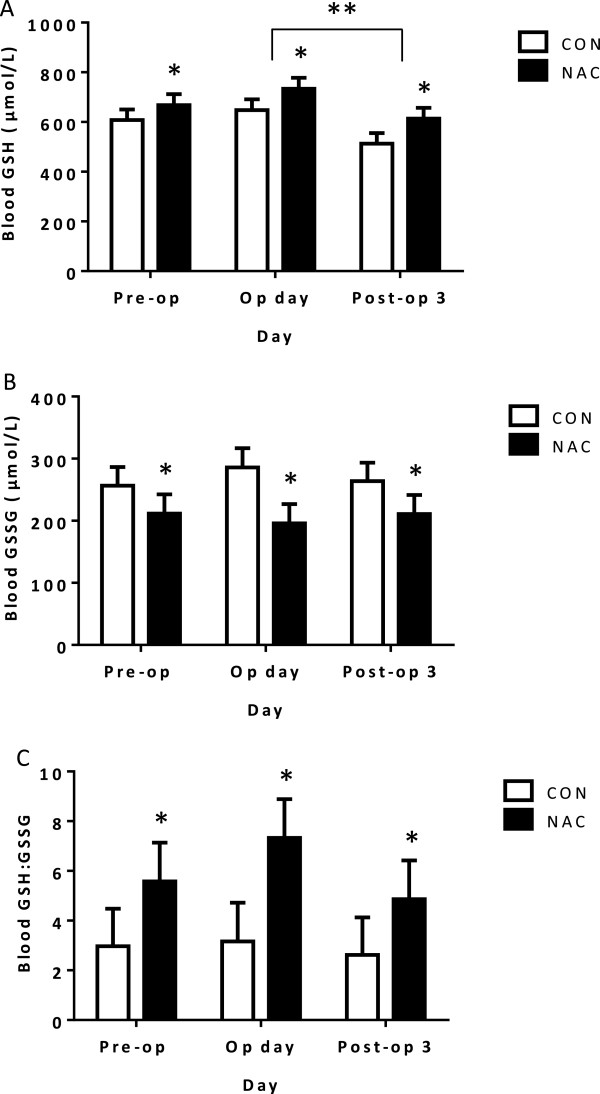
**Data presented as mean ± SEM.** CON n = 17, NAC n = 16. **A**: Blood reduced glutathione (GSH) levels. *Indicates the significant effect of NAC treatment when compared to control group (P = 0.023). **Indicates the significant effect of day. Tukey’s post-hoc test showed a significant difference between operation day and post-operational third day on blood GSH levels in both study groups (P = 0.016). **B**: Blood oxidised glutathione (GSSG) levels. *Indicates the significant effect of NAC treatment when compared to control group (P = 0.014). No significant effect of day was found on GSSG levels. **C**: *Indicates the significant effect of NAC treatment when compared to control group (P = 0.019). No significant effect of day was found on GSH/GSSG ratio.

NAC supplementation also had remarkable influence on the variables associated with glutathione redox cycle (Figure 2). Although there was a marked decrease in whole blood GSH levels during post-operation, when compared to operation day in both groups (690.78 ± 30.99 µmol/L, operation day versus 563.38 ± 30.53 µmol/L, post-operational third day, P = 0.016), NAC treatment resulted in significantly increased GSH levels when compared to controls (589.58 ± 24.80 µmol/L, CON versus 671.76 ± 25.30 µmol/L, NAC, P = 0.023). Whilst whole blood GSH levels increased in the NAC group, GSSG levels decreased significantly (268.74 ± 17.43 µmol/L, CON versus 206.07 ± 17.78 µmol/L, NAC, P = 0.014) and independently of the day (Figure 2). These effects of NAC treatment on GSH and GSSG levels were reflected in the GSH:GSSG ratio, which was higher in patients receiving NAC treatment (2.92 ± 0.88 µmol/L, CON versus 5.92 ± 0.90 µmol/L, NAC, P = 0.019) (Figure 2).

**Table 2:** **Oxidant and antioxidant parameters**

	Group	Day				
		Pre-op	Op day	Post-op 1	Post-op 3	Post-op 5
MDA (µmol/L)*	CON	0.45 ± 0.03	0.45 ± 0.03	0.45 ± 0.03	0.44 ± 0.03	0.43 ± 0.03
	NAC	0.35 ± 0.03	0.35 ± 0.03	0.37 ± 0.03	0.36 ± 0.03	0.36 ± 0.03
Nitrate (µmol/L)^*^	CON	293.58 ± 69.22	287.16 ± 69.22	262.63 ± 69.22	381.18 ± 69.22	529.09 ± 71.35
	NAC	197.44 ± 71.35	211.11 ± 71.35	285.81 ± 71.3	242.49 ± 71.35	274.01 ± 71.35
Nitrite (µmol/L)	CON	8.61 ± 2.38	11.28 ± 2.37	9.85 ± 2.37	14.96 ± 2.37	9.45 ± 2.45
	NAC	9.39 ± 2.45	8.13 ± 2.45	10.91 ± 2.45	9.83 ± 2.45	8.24 ± 2.45
Vitamin A (µmol/L)	CON	1.22 ± 0.09^a^	0.76 ± 0.09^b^	0.51 ± 0.09^b^	0.64 ± 0.09^b^	0.88 ± 0.09^b^
	NAC	1.43 ± 0.09^a^	0.89 ± 0.10^b^	0.64 ± 0.09^b^	0.64 ± 0.09^b^	0.77 ± 0.10^b^
Vitamin C (mg/L)^‡^	CON	2.66 ± 0.22	2.68 ± 0.23	2.65 ± 0.22	2.48 ± 0.22	2.49 ± 0.24
	NAC	2.29 ± 0.23	2.26 ± 0.23	2.07 ± 0.23	1.99 ± 0.23	1.96 ± 0.23
Vitamin E (µmol/L)	CON	29.24 ± 1.65	20.54 ± 1.65	16.04 ± 1.65	18.97 ± 1.65	21.22 ± 1.76
	NAC	34.86 ± 1.70	21.03 ± 1.70	16.30 ± 1.70	18.20 ± 1.70	20.09 ± 1.70

Data presented as mean ± SEM.

*Plasma malondialdehyde level was influenced by NAC treatment (P < 0.001).

^*^Urinary nitrate level was influenced by NAC treatment (P = 0.016).

^‡^Plasma vitamin C level was influenced by NAC treatment (P = 0.001).

^a,b^Mean values with unlike superscript letters in the same row were significantly different (P < 05).

One of the aims of the present was to assess whether NAC treatment had any impact upon antioxidant vitamins. In this context, plasma vitamin A, C, and E levels were analyzed. Although oxidant parameters were influenced by NAC treatment from the early phase of the study, antioxidant vitamins did not exhibit the same trend (Table 2). Plasma vitamin A levels were significantly higher during the pre-operational period in both groups (1.32 ± 0.07 µmol/L, pre-operational day versus 0.82 ± 0.07 µmol/L, operation day, 0.58 ± 0.07 µmol/L, post-operational first day, 0.64 ± 0.07 µmol/L, post-operational third day, 0.83 ± 0.07 µmol/L, post-operational fifth day, P < 0.001). Interestingly, vitamin C level was found to be significantly lower in the NAC group, irrespective of the study days (2.59 ± 0.10 mg/L, CON versus 2.11 ± 0.10 mg/L, NAC, P = 0.001) and vitamin E levels were similar between all groups (Table 2).

## Discussion

This study aimed to examine the influence of NAC supplementation on plasma amino acid levels, as well as antioxidant and oxidant parameters, in cancer patients undergoing major abdominal surgery. The results demonstrated that NAC treatment, starting from 2 days before the operation day and lasting until the post-operational fifth day, influence some important markers of oxidative stress in these patients. MDA, which is a crucial indicator of lipid peroxidation, was 18.18% lower in the NAC treated group when compared to controls. Similarly, nitrate levels, which can be considered as an oxidative stress biomarker in urine, decreased 30.95% in the NAC group. Given that intracellular GSSG accumulates and the GSH:GSSG ratio decreases under increased levels of oxidative stress, increased GSH:GSSG ratio implies an improved cellular oxidative capacity [6]. Here we found that the GSH:GSSG ratio was 50.68% greater in the NAC group than controls.

Surgery initiates a wide spectrum of suboptimal alterations in body homeostasis and is closely associated with several complications of surgical stress, of which exposure to increased oxidative stress is considered as an important component [25]. From this perspective, improved antioxidant mechanisms may help to combat oxidative stress. Although the current study demonstrated diminished levels of oxidant stress factors, antioxidant factors such as vitamin A, C, and E did not exhibit improvement. In fact, vitamin C level was found to be lower in the NAC group. To date, only a few studies have examined the effects of NAC and antioxidant vitamins on disease and reported beneficial effects of these combined therapies [26, 27], however, the principal reason behind the lower or unaltered levels of these vitamins in the current study remains unknown. A recent study has shown that NAC administration during high-intensity exercise did not change blood glutathione levels but diminished lipid peroxidation [28], and there is also evidence regarding the altered pre-operative and post-operative total oxidative stress and total antioxidant capacities following NAC treatment [29]. Therefore total oxidative stress and total antioxidant capacities should be measured in future studies to elucidate the antioxidant mechanisms that are associated with NAC action. In addition, cancer patients tend to have lower circulating concentrations of antioxidant vitamins, either due to altered nutritional status, increased catabolic processes, or inflammatory response [30]. Indeed, the current study revealed that all plasma antioxidant vitamin levels were lower in the NAC group than in healthy individuals. To further investigate the clinical relevance of this finding, future studies should focus on antioxidant vitamin supplementation together with NAC, and seek the oxidant status of these patients.

NRI is a useful tool for the early identification of nutritional depletion in hospitalised patients [31]. It was considered that determining the nutritional status of patients before surgery was needed to address the increased risk of malnutrition related disorders, and that NRI data reflected a mild risk of malnutrition in the current study. This situation emphasizes the importance of pre-operative care strategies in order to combat malnutrition and post-operational morbidity and mortality, since these factors were shown to be related [32]. Since hospital malnutrition is one of the most important challenges in clinical practice, novel strategies should be developed to avoid the progress of malnutrition in cancer patients undergoing surgery. A study which used NAC infusion at a rate of 0.3 mg/kg/min intravenously during surgery and 0.2 mg/kg/min for 24 h during post-operation showed a significantly shorter period in the intensive care unit length of stay [33]. Whereas a prophylactic high dose oral of NAC was reported to be ineffective on post-operative hospital stay after heart surgery [18]. The current study also did not exhibit a significant effect on length of stay in hospital. In fact, despite a non-significant outcome, post- or total length of stay data at hospital appeared to be increased in males. The reason of this outcome can be attributable to specific gender differences rather than an experimental effect, since some studies indicated that gender may play a deterministic role in duration of length of stay in hospital [34, 35]. Future studies should examine whether these differences can be attributable to specific responses to the treatment, preventable complications, or bias. In addition, the effect of NAC supplementation on hospital care should be considered in future studies, since NAC may exert a protective effect through shortening the length of stay, and lowering malnutrition rate and morbidity in the post-operative period.

The therapeutic feature of antioxidants relies on their capacity to cross the cell membranes [12]. When NAC is administered through oral or intravenous routes, it undergoes N-deacetylation [12]. There is some conflicting data about the mechanisms of the antioxidant action of NAC as it has not yet been clearly shown whether the effectiveness of NAC should be attributed to its direct involvement in synthesis of intracellular GSH after N-deacetylation, its ability to reduce extracellular cystine to cysteine, or its direct chelating property to free radicals [13]. The current study demonstrated a profound increase in GSH levels in the NAC treated group, as in other studies [13, 36]. These data clearly indicate the impact of NAC supplementation on GSH synthesis. However, the effect of NAC supplementation on cystine to cysteine reduction is not clear since the cystine data introduced in this study did not distinguish the reduced cysteine and cystine. It is known that more than 90% of the total soluble cysteine in plasma is in the oxidized cystine form [37]. In another study, in which the kinetics of uptake and deacetylation of NAC in erythrocytes was investigated, NAC was found to replace a cysteine with a cystine molecule and enhance plasma free cysteine levels [38]. The current study exhibited a significant increase in cystine levels following NAC supplementation, especially after the operation day. Since the cysteine levels were undetected, the interpretation of this outcome becomes complicated. However, when the oxidative milieu of blood and short half-life of NAC is taken into account, rapid oxidation of cysteine to cystine should also be considered [39]. However, it should also be considered that the amount of available intracellular cysteine is the limiting factor in GSH synthesis. Therefore, our finding of increased GSH in the NAC treated group may suggest that the amount of NAC taken was sufficient to induce GSH synthesis in these patients. Moreover, continuous infusion of NAC through total parenteral nutrition may help to reduce the risk of oxidative injury. Further research should focus on the interactions between NAC and related biomarkers to reinforce current knowledge about the mechanisms of NAC.

In disease and trauma, adequacy of specific amino acids becomes an important issue. It is well-known that requirements of the amino acids may change under traumatic conditions [40]. Beyond their common functions, specific amino acid supplementation in total parenteral nutrition was shown to induce beneficial effects in operative cancer patients [41, 42]. Therefore, one of the aims of the current was to determine plasma amino acid levels along with NAC supplementation. Despite the clear effect of NAC on plasma cysteine, as discussed above, no major effect of NAC was found on other plasma amino acid levels. In fact, operation day appeared to have a greater impact on plasma amino acid levels. A recent study that investigated the changes in plasma BCAA and glutamine concentrations in operative gastrointestinal cancer patients indicated that the fall in BCAA levels were partially prevented by total parenteral nutrition [43]. Our results did not exhibit such an effect on BCAA, whereas AAA appeared to remain at higher levels during the treatment. Further studies investigating the influence of total parenteral nutrition on plasma protein and amino acid levels in surgery patients should be performed.

N-acetyl cysteine supplementation was associated with pro-oxidant effects in experimental models, which caused concerns about its application in clinical practice when there is a lack of significant oxidative stress factor [12]. Certainly, the amount of NAC used is the determinative factor for the pro-oxidant effects. In the current study, NAC was infused at a rate of 300 mg in every 6 h (i.e., 1,200 mg/day). This amount is a low to intermediate level when compared to other studies in the literature [13, 18]. In another study, even a low dose of NAC supplementation, which was 2 to 4 mmol/l in cardioplegia solution, significantly reduced the MDA levels [44]. In a study in which the pro-oxidant and deleterious effects of NAC were shown in healthy individuals, NAC was used up to 2,400 mg over longer periods [45]. Therefore, using 1,200 mg of NAC/day provided a safe and clinically relevant dose which could be considered in further studies and applications.

This study examined the hypotheses that NAC may improve oxidant and antioxidant parameters in cancer patients undergoing major abdominal surgery, and the data generated supports the assertion that NAC may provide beneficial effects in these patients. Despite these improved parameters, it should be taken into account that the present study has several limitations. One of the most important limitations is the lack of follow-up of patients. Monitoring the prognosis of patients and other physical outcomes would provide a complete understanding of the clinical consequences. Therefore, future studies should acknowledge the long-term and solid effects of NAC supplementation on prognosis, recovery, and survival rates of the patients in addition to the length of hospitalization. Secondly, large-scale population studies are needed to generalise the beneficial effects of NAC on surgery patients. A recent systematic review that aimed to analyse the effectiveness of pharmacological modulation of oxidative stress in surgery related interventions of animal models, reported that NAC was effective at reducing oxidative stress markers [46]. Although the exact mechanisms of this action remains to be understood completely, these results indicate the clinical relevance of translating this approach to human studies. Consequently, supporting oxidative defence mechanisms in surgery patients should be evaluated within the limits of ordinary clinical practice, yet these practices may help to improve normal body homeostasis in these patients.
